# Serum α-Tocopherol and γ-Tocopherol Concentrations and Prostate Cancer Risk in the PLCO Screening Trial: A Nested Case-Control Study

**DOI:** 10.1371/journal.pone.0040204

**Published:** 2012-07-05

**Authors:** Stephanie J. Weinstein, Ulrike Peters, Jiyoung Ahn, Marlin D. Friesen, Elio Riboli, Richard B. Hayes, Demetrius Albanes

**Affiliations:** 1 Nutritional Epidemiology Branch, Division of Cancer Epidemiology and Genetics, National Cancer Institute, National Institutes of Health, Department of Health and Human Servies, Bethesda, Maryland, United States of America; 2 Cancer Prevention Program, Fred Hutchinson Cancer Research Center, Seattle, Washington, United States of America; 3 Department of Epidemiology, School of Public Health, University of Washington, Seattle, Washington, United States of America; 4 Division of Epidemiology, Department of Population Health, New York University School of Medicine, New York, New York, United States of America; 5 Department of Environmental Health Sciences, Johns Hopkins Bloomberg School of Public Health, Baltimore, Maryland, United States of America; 6 Department of Epidemiology and Public Health, Imperial College, London, United Kingdom; Sookmyung Women’s University, Republic of Korea

## Abstract

**Background:**

Vitamin E compounds exhibit prostate cancer preventive properties experimentally, but serologic investigations of tocopherols, and randomized controlled trials of supplementation in particular, have been inconsistent. Many studies suggest protective effects among smokers and for aggressive prostate cancer, however.

**Methods:**

We conducted a nested case-control study of serum α-tocopherol and γ-tocopherol and prostate cancer risk in the Prostate, Lung, Colorectal and Ovarian Cancer Screening Trial, with 680 prostate cancer cases and 824 frequency-matched controls. Multivariate-adjusted, conditional logistic regression models were used to estimate odds ratios (OR) and 95% confidence intervals (CIs) for tocopherol quintiles.

**Results:**

Serum α-tocopherol and γ-tocopherol were inversely correlated (r = −0.24, p<0.0001). Higher serum α-tocopherol was associated with significantly lower prostate cancer risk (OR for the highest vs. lowest quintile = 0.63, 95% CI 0.44–0.92, p-trend 0.05). By contrast, risk was non-significantly elevated among men with higher γ-tocopherol concentrations (OR for the highest vs. lowest quintile = 1.35, 95% CI 0.92–1.97, p-trend 0.41). The inverse association between prostate cancer and α-tocopherol was restricted to current and recently former smokers, but was only slightly stronger for aggressive disease. By contrast, the increased risk for higher γ-tocopherol was more pronounced for less aggressive cancers.

**Conclusions:**

Our findings indicate higher α-tocopherol status is associated with decreased risk of developing prostate cancer, particularly among smokers. Although two recent controlled trials did not substantiate an earlier finding of lower prostate cancer incidence and mortality in response to supplementation with a relatively low dose of α-tocopherol, higher α-tocopherol status may be beneficial with respect to prostate cancer risk among smokers. Determining what stage of prostate cancer development is impacted by vitamin E, the underlying mechanisms, and how smoking modifies the association, is needed for a more complete understanding of the vitamin E-prostate cancer relation.

## Introduction

Vitamin E compounds are thought to have potential prostate cancer preventive effects, but randomized controlled trials have been inconsistent. Earlier findings of a one-third reduction in prostate cancer incidence in response to daily supplementation with 50 mg (50 IU) of α-tocopherol from the Alpha-Tocopherol, Beta-Carotene Cancer Prevention (ATBC) Study of smokers [1] were not substantiated by two recent trials, the Selenium and Vitamin E Cancer Prevention Trial (SELECT) and the Physicians’ Health Study II Randomized Controlled Trial (PHS-II), which included primarily nonsmokers and tested either a 400 IU daily dose [2], [3] or a 400 IU alternate day dose [4] of vitamin E (α-tocopherol). In addition to the differences in smoking status and supplementation dose across the three trials, the SELECT protocol included pre-randomization (for exclusion) prostate cancer screening which resulted in a preponderance of stage Ia prostate cancers being diagnosed during the trial [2]. Observational data also suggest a vitamin E-prostate cancer-smoking interaction, with a beneficial association for supplemental vitamin E or higher tocopherol status in smokers and for aggressive, and not non-aggressive, disease [5]–[13].

Given the high incidence of prostate cancer in the U.S. and elsewhere, the biological plausibility that vitamin E could impact cancer risk through several mechanisms [14]–[18], and the conflicting observational and controlled trial data, further examination of the vitamin E - prostate cancer relationship is needed. To this end, we conducted a prospective nested case-control study of serum concentrations of the two major tocopherols, α- and γ-, in relation to prostate cancer risk in the Prostate, Lung, Colorectal, and Ovarian Cancer Screening Trial (PLCO). Key *a priori* aims of our analysis included examination of the serologic dose-risk relation, the relative impact of the two vitamin E congeners, and effect modification by smoking and disease aggressiveness.

## Methods

### Ethics Statement

The study was approved by the institutional review boards of the U.S. National Cancer Institute and the ten PLCO screening centers, and participants provided written informed consent.

### Study Population

We conducted a case-control study nested within the PLCO Screening Trial, an on-going community-based study evaluating the effectiveness of cancer screening tests on site-specific cancer mortality. Participants (ages 55–74) were recruited from ten centers in the United States (Birmingham, AL; Denver, CO; Detroit, MI; Honolulu, HI; Marshfield, WI; Minneapolis, MN; Pittsburgh, PA; Salt Lake City, UT; St Louis, MO; and Washington, DC) between September 1993 and June 2001.

Men randomized to the screening arm of the trial were offered prostate cancer screening by serum prostate-specific antigen (PSA) and digital rectal examination (DRE) at entry and annually for 5 and 3 years, respectively. Men with a PSA test result >4 ng/ml or a DRE exam suspicious for prostate cancer were referred to their medical-care providers for follow-up. Trial participants were asked to provide information regarding recent cancer diagnoses through annual mailed follow-up questionnaires, and medical and pathologic records related to diagnostic follow-up of prostate cancer were obtained by study personnel from medical providers. Periodic searches of the National Death Index were also conducted and death certificates and medical and pathology records related to death were obtained. Data were abstracted by trained medical record specialists.

### Data Collection

At enrollment, all participants were asked to complete a risk factor questionnaire including age, ethnicity, education, occupation, smoking history, personal and family medical history, use of selected drugs, recent history of screening exams, and prostate related health factors. In addition, usual dietary intake over the 12 months prior to enrollment was assessed with a 137-item food frequency questionnaire (http://www.cancer.gov/prevention/plco/DQX.pdf). Sex- and age-specific portion size and nutrient values were quantified [19]. Total vitamin and mineral intake was calculated by adding dietary and supplemental intake. Non-fasting blood samples were obtained at baseline and in subsequent screening exams from participants in the screening arm [20]. All samples were shipped overnight to a central biorepository and stored at –70^o^C.

### Case Identification and Control Selection

This prostate cancer nested case-control set has been previously described [21]. Briefly, the study included men randomized to the screening arm of the trial, whose first valid prostate cancer screen (PSA test or DRE) was before October 1, 2001. All men were followed from their initial screen to the earliest of: prostate cancer diagnosis, loss-to-follow-up, death, or censor date (October 1, 2001). Cases were defined as non-Hispanic white participants diagnosed with adenocarcinoma of the prostate at least 1 year after blood draw (n = 803). Aggressive cases were defined as those with stage III or IV of the tumor-node-metastasis staging system, as defined by the American Joint Committee on Cancer [22], or Gleason score ≥7. Controls (n = 949) were selected by incidence-density sampling [23] with a case-control ratio of 1∶1.2, frequency-matched by age (5-year intervals), time since initial screening (1-year time windows), and year of blood collection. Baseline serum was available for 692 of these cases and 844 controls. We excluded subjects with missing serum cholesterol data, resulting in an analytic set of 680 cases (including 267 aggressive cases) and 824 controls. In a sub-sample of 46 controls, we measured serum tocopherols in samples drawn at baseline and one year follow-up.

### Laboratory Analyses

Serum concentrations of α- and γ-tocopherol were determined using reversed-phase high-performance liquid chromatography, with ultraviolet detection [24]. Cholesterol was determined enzymatically using a Hitachi 912 autoanalyzer with a standard procedure at 37°C. Batches of serum samples were organized to include cases, their matched controls, and randomly inserted blinded quality controls. The overall coefficients of variation estimated from the 171 of the latter samples were 5.8% for α-tocopherol and 8.9% for γ-tocopherol. Serum retinol, β-carotene, and lycopene were previously measured [21], [24].

### Statistical Analyses

Case and non-case characteristics were compared using t-tests and chi-square tests, for continuous and categorical variables, respectively. Spearman correlations were calculated for tocopherol measurements among the controls taken at two time points, as well as for each tocopherol with age, body mass index (BMI), and several dietary and serum factors. Partial Spearman correlations were used to adjust for factors such as month of blood draw, serum cholesterol concentration, smoking, BMI, age, and energy intake. Conditional logistic regression models were used to estimate odds ratios (OR) and 95% confidence intervals (CIs) for the association between prostate cancer and serum tocopherols. Quintile categories of the nutrients were created based on the distribution among the controls, and entered into the models as indicator variables with the lowest quintile as the referent category. Quintile categories were also calculated separately for analyses stratified on vitamin E supplement dosage, categorized as ≤50 IU/day (defined for purposes in this manuscript as “non-users of vitamin E supplements”) vs. >50 IU/day (defined as “vitamin E supplement users”) from either individual or multivitamin supplements. This categorization was selected because the 50 IU/day supplement in the ATBC Study resulted in a significant increase in serum α-tocopherol [25], and because PLCO participants reported vitamin E intake from multivitamin supplements only of greater than 50 IU/day. Tests for linear trend were obtained by assigning to each nutrient quintile the median value and treating this as a continuous variable. The multivariate models were conditioned on the matching factors (age, time since initial screening, and year of blood draw), and adjusted for serum cholesterol, serum β-carotene, and study center. The following were not confounders in our sample (i.e., when adding each to the model, a <10% change in any of the nutrient coefficients resulted): height, weight, BMI, smoking status, physical activity, educational attainment, marital status, aspirin and ibuprofen use, history of diabetes, history of benign prostatic hyperplasia, family history of prostate cancer, average number of prostate screens (PSA or DRE) per year, serum selenium, month of blood draw, vitamin supplement use, and intakes of total energy, total fat, fruits, vegetables, alcohol, red meat, heterocyclic amine from meat (2-amino-1-methyl-6-phenylimidazo[4,5-b]pyridine), lycopene, vitamin C, vitamin E, and calcium. Results are also presented stratified by stage (non-aggressive/aggressive as described above) and smoking status (never-smokers, current smokers, current smokers combined with former smokers who quit <10 years ago, and former smokers who quit ≥10 years ago). Additional subgroup analyses were based on high/low (medians) of age, BMI; serum cholesterol, selenium, and β-carotene; dietary selenium, vitamin C, vitamin E (dietary only and total); vitamin E supplement dose; and follow-up time (1–2 yrs vs. 3 or more years). All stratified models were run using unconditional logistic regression. Multiplicative interactions were tested statistically by comparing models with and without a cross-product interaction term (tocopherol quintiles crossed with the effect modifier split at the median) using the log-likelihood ratio test. Statistical analyses were performed using SAS software version 9.2 (SAS Institute, Inc., Cary, North Carolina) and all p-values were two-sided.

## Results

Selected baseline characteristics of cases and controls are shown in Table 1. Cases were more likely to have a history of benign prostatic hyperplasia or familial prostate cancer, and less likely to take aspirin more than once per week. Cases tended to smoke less than controls, although this difference was not statistically significant. Average daily intake of dietary and total (diet plus supplements) vitamin E did not differ by case status. These patterns were similar when aggressive cases were compared with controls, with the exception that aggressive cases had a significantly lower history of diabetes compared with controls (p = 0.04). As was the situation for all cases, aggressive cases tended to smoke less than controls, but this difference was not statistically significant (p = 0.16). Reported doses of supplemental vitamin E ranged from 4 to 1060 IU/day, but 30 IU/day (from a multivitamin supplement), 400 IU/day (from an individual vitamin E supplement), and 430 IU/day (from a combination of a multivitamin and an individual supplement) were most prevalent −27%, 16% and 17%, respectively, of controls who took vitamin E. Approximately 30% of controls reported consuming 50 IU/day or greater of vitamin E from any type of supplement.

**Table 1:** Selected baseline characteristics by case or control status^a^, PLCO Study.

Characteristic	Cases (n = 680)	Controls (n = 824)	P^b^
Age at study entry, y	64.9 (4.9)	64.7 (4.8)	0.36
Education (% college graduate)	44.0	42.3	0.63
Average number of prostate screens/yr^c^	0.95 (0.11)	0.96 (0.10)	0.34
History of benign prostatic hyperplasia, %	32.2	25.2	0.003
Family history of prostate cancer, %	11.1	5.5	<0.0001
History of diabetes, %	6.0	8.0	0.28
Height, cm	178 (6)	178 (7)	0.10
Weight, kg	86.4 (13.0)	86.6 (13.6)	0.78
Body mass index, kg/m^2^	27.1 (3.6)	27.4 (3.9)	0.13
Vigorous physical activity, h/wk (%)			0.11
<1	27.0	29.8	
1–3	44.3	38.8	
≥4	28.8	31.4	
Smoking history, %			0.09
Never-smoker	36.3	30.2	
Current smoker	6.8	9.2	
Former smoker (quit <10 yrs ago)	8.3	8.4	
Former smoker (quit ≥10 yrs ago)	40.9	43.0	
Pipe/cigar only	7.7	9.3	
Aspirin use, ≥1 times/week, %	43.1	48.3	0.04
Dietary intake/day			
Energy, kcal	2384 (878)	2343 (923)	0.40
Total fat, g	80 (37)	79 (39)	0.56
Calcium, mg	1195 (561)	1162 (598)	0.27
Vitamin D, IU	424 (311)	417 (331)	0.71
Vitamin E, mg	9.5 (4.6)	9.4 (4.8)	0.49
Vitamin E (diet and supplements), mg	67.6 (109.0)	73.3 (108.8)	0.32
Supplemental vitamin E^d^ ≥50 IU/day	26.9	30.5	0.13
Serum biochemical measures			
α-Tocopherol, mg/L	19.0 (9.8)	19.0 (9.5)	0.92
γ-Tocopherol, mg/L	3.2 (2.0)	3.3 (2.0)	0.59
α-Tocopherol:γ-tocopherol molar ratio	10.9 (15.5)	10.6 (13.2)	0.72
β-Carotene, µg/dL	22.8 (23.5)	20.9 (22.4)	0.11
Retinol, µg/dL	70.9 (23.7)	71.9 (24.6)	0.42
Lycopene, µg/dL	67.2 (31.9)	65.9 (31.1)	0.45
Cholesterol, mmol/L	6.08 (1.92)	6.10 (1.94)	0.80

a Data are mean (standard deviation), or percents.

b P-value based on t-tests or chi-square tests, for continuous and categorical variables, respectively.

c Average number of prostate cancer screening examinations (PSA or DRE) up to diagnosis of prostate cancer (cases) or selection as a control.

d Including from both single and multivitamin supplements.

Among controls, serum α-tocopherol and γ-tocopherol were weakly inversely correlated (r = −0.24, p<0.0001; r = −0.39, p<0.0001 with adjustment for serum total cholesterol). Both tocopherols were strongly associated with the α-tocopherol:γ-tocopherol molar ratio and weakly associated with serum β-carotene and BMI, but in opposite directions, and weakly correlated with serum cholesterol, retinol, and lycopene (Table 2). Total vitamin E intake, but not dietary vitamin E intake alone, was positively correlated with serum α-tocopherol and inversely correlated with serum γ-tocopherol; adjustment for month of blood draw, serum cholesterol concentration, smoking, BMI, age, and energy intake had no material impact on these relations (i.e., with adjustment, r = 0.58 for α-tocopherol and −0.56 for γ-tocopherol). Vitamin E supplement use was associated with higher median serum α-tocopherol (14.8 mg/L for non-users vs. 23.7 mg/L for users, p<0.0001) and lower serum γ-tocopherol (3.5 mg/L for non-users vs. 1.4 mg/L for users, p<0.0001). In a sub-sample of 46 controls, two measurements of serum α-tocopherol and γ-tocopherol from baseline and one year follow-up were well-correlated (r = 0.58 and r = 0.80, respectively; both p<0.0001, data not shown). Tocopherol concentrations did not differ by smoking status. For example, median serum α-tocopherol was 16.1, 17.0, 17.2 and 16.8 mg/L for never smokers, former smokers (quit ≥10 years ago), former smokers (quit <10 years ago, and current smokers, respectively (p = 0.28).

**Table 2:** Correlations between baseline characteristics and α-tocopherol and γ-tocopherol among controls, PLCO Study.

Characteristic	α-Tocopherol		γ-Tocopherol	
	r	p-value	R	p-value
Age, y	−0.0002	0.99	−0.08	0.03
Body mass index, kg/m^2^	−0.08	0.02	0.19	<0.0001
Vitamin E intake (diet), mg/day	0.02	0.57	−0.06	0.09
Vitamin E intake (diet and supplements), mg/day	0.49	<0.0001	−0.54	<0.0001
Serum biochemical measures				
α-Tocopherol, mg/L	–	–	−0.24	<0.0001
γ-Tocopherol, mg/L	−0.24	<0.0001	–	–
α-Tocopherol:γ-tocopherol molar ratio	0.62	<0.0001	−0.88	<0.0001
β-Carotene, µg/dL	0.36	<0.0001	−0.22	<0.0001
Retinol, µg/dL	0.46	<0.0001	0.12	0.0004
Lycopene, µg/dL	0.24	<0.0001	0.14	<0.0001
Cholesterol, mmol/L	0.38	<0.0001	0.29	<0.0001

Higher serum α-tocopherol was associated with statistically significantly lower risk of prostate cancer (OR = 0.63, 95% CI 0.44–0.92, for the highest vs. lowest quintile, p-trend 0.05, Table 3). By contrast, there was no clear association with serum γ-tocopherol, although elevated risk was suggested for the four higher quintiles, but with no evidence of dose-response. The patterns were similar when mutually-adjusting for both tocopherols (data not shown). The molar ratio of α-tocopherol to γ-tocopherol was not related to risk of prostate cancer, although risk was significantly elevated for the second quintile (Table 3). The exclusion of vitamin E supplement users (50 IU or greater/day) resulted in an attenuated risk for α-tocopherol [OR = 0.87 (95% CI 0.55–1.38) for the highest quintile (>19.9 mg/L, median = 23.3 mg/L) vs. lowest quintile (≤11.4 mg/L, median = 10.0 mg/L)] and no association for γ-tocopherol [OR = 1.15 (95% CI 0.74–1.79) for the highest quintile (>5.32 mg/L, median = 6.52) vs. lowest quintile (≤2.28 mg/L, median = 1.67 mg/L)]. However, among supplemental vitamin E users, risk appeared lower for higher α-tocopherol [OR = 0.54, 95% CI 0.26–1.01 for the highest quintile (>33.2 mg/L, median = 40.7 mg/L) vs. lowest quintile (≤17.0 mg/L, median = 14.2 mg/L)] with no association for γ-tocopherol [OR = 0.97 (95% CI 0.47–2.01) for the highest quintile (>2.82 mg/L, median = 3.64) vs. lowest quintile (≤0.83 mg/L, median = 0.65 mg/L)]. Post-hoc joint classification using as the reference category men at elevated risk based on both α-tocopherol and γ-tocopherol (i.e., in quintile 1 of α-tocopherol and quintiles 2–5 of γ-tocopherol) revealed an OR of 0.53 (95% CI 0.36–0.77) for men with the lowest risk profile (i.e. in quintiles 2–5 of α-tocopherol and quintile 1 of γ-tocopherol), and an OR = 0.74 (95% CI 0.55–1.00) for the two intermediate risk categories combined (p-trend = 0.001).

**Table 3:** Association between baseline serum α-tocopherol, γ-tocopherol, and the α-tocopherol:γ-tocopherol molar ratio and risk of prostate cancer, PLCO Study.

	Serum tocopherol quintiles						
	1	2	3	4	5	*P*_trend_	
α-Tocopherol, mg/L	≤12.3	>12.3 & ≤15.0	>15.0 & ≤18.7	>18.7 & ≤24.5	>24.5		
Median, mg/L	10.4	13.8	16.7	20.6	30.6		
Cases/controls, N	155/165	126/165	139/165	131/165	129/164		
OR^a^ (95% CI)	1.00 (reference)	0.73 (0.52–1.03)	0.75 (0.53–1.06)	0.67 (0.47–0.96)	0.63 (0.44–0.92)	0.05	
γ-Tocopherol, mg/L	≤1.38	>1.38 & ≤2.49	>2.49 & ≤3.48	>3.48 & ≤4.78	>4.78		
Median, mg/L	0.96	1.94	3.00	4.05	5.83		
Cases/controls, N	116/165	151/165	165/165	125/165	123/164		
OR^a^ (95% CI)	1.00 (reference)	1.52 (1.08–2.13)	1.63 (1.16–2.30)	1.34 (0.92–1.97)	1.35 (0.92–1.97)	0.41	
α-Tocopherol: γ-tocopherol molar ratio	≤2.97	>2.97 & ≤4.16	>4.16 & ≤6.32	>6.32 & ≤15.83	>15.83		
Median	2.53	3.50	5.00	9.41	28.1		
Cases/controls, N	103/165	161/165	145/165	145/165	126/164		
OR^a^ (95% CI)	1.00 (reference)	1.46 (1.04–2.05)	1.24 (0.87–1.77)	1.17 (0.82–1.68)	0.96 (0.66–1.39)	0.09	

a Odds ratios based on conditional logistic regression (conditioned on age, time since initial screening, and year of blood draw) and adjusted for study center, serum cholesterol and serum β-carotene.

Serum α-tocopherol was inversely associated with both non-aggressive and aggressive prostate cancer, although the odds ratios for each quintile were stronger for aggressive disease (Table 4). By contrast, elevated risk for higher serum γ-tocopherol appeared stronger for non-aggressive disease, with a threshold above the lowest quintile and confidence intervals for three OR’s excluding 1.0, but with no significant trend. There was no clear relationship between the molar ratio of the two tocopherols and risk of either non-aggressive or aggressive prostate cancer (data not shown). When comparing the top four quintiles to the lowest quintile of α-tocopherol, the odds ratios for non-aggressive and aggressive disease were 0.76 (95% CI 0.55–1.06, p = 0.11) and 0.67 (95% CI 0.46–0.98, p = 0.04), respectively. Similar risks for γ-tocopherol were OR = 1.64 (95% CI 1.16–2.32, p = 0.01) and OR = 1.17 (95% CI 0.80–1.72, p = 0.41), and for the α-tocopherol:γ-tocopherol ratio were OR = 1.19 (95% CI 0.86–1.66, p = 0.30) and OR = 1.41 (95% CI 0.92–2.16, p = 0.12).

**Table 4:** Association between baseline serum α-tocopherol and γ-tocopherol and risk of prostate cancer, stratified by disease stage and grade, PLCO Study.

	Serum tocopherol quintiles						
	1	2	3	4	5	*P*_trend_	
α-Tocopherol, mg/L	≤12.3	>12.3 & ≤15.0	>15.0 & ≤18.7	>18.7 & ≤24.5	>24.5		
Median, mg/L	10.4	13.8	16.7	20.6	30.6		
Non-aggressive							
Cases/controls, N	90/165	79/165	86/165	78/165	80/164		
OR^a^ (95% CI)	1.00 (reference)	0.78 (0.52–1.15)	0.83 (0.56–1.24)	0.70 (0.46–1.07)	0.71 (0.46–1.09)	0.18	
Aggressive^b^							
Cases/controls, N	65/165	47/165	53/165	53/165	49/164		
OR^a^ (95% CI)	1.00 (reference)	0.67 (0.42–1.06)	0.72 (0.45–1.14)	0.63 (0.39–1.03)	0.65 (0.39–1.07)	0.19	
γ-Tocopherol, mg/L	≤1.38	>1.38 & ≤2.49	>2.49 & ≤3.48	>3.48 & ≤4.78	>4.78		
Median, mg/L	0.96	1.94	3	4.05	5.83		
Non-aggressive							
Cases/controls, N	63/165	93/165	101/165	69/165	87/164		
OR^a^ (95% CI)	1.00 (reference)	1.69 (1.13–2.54)	1.90 (1.27–2.87)	1.30 (0.84–2.00)	1.65 (1.06–2.56)	0.24	
Aggressive^b^							
Cases/controls, N	53/165	58/165	64/165	56/165	36/164		
OR^a^ (95% CI)	1.00 (reference)	1.17 (0.74–1.84)	1.30 (0.82–2.04)	1.21 (0.75–1.94)	0.89 (0.52–1.52)	0.71	

a Odds ratios are based on unconditional logistic regression, adjusted for study center, serum cholesterol, serum β-carotene, age, time since initial screening, and year of blood draw.

b Aggressive cases were defined as stage III or IV, or Gleason score ≥ 7.

Analyses stratified by smoking status showed lower risk with increasing serum α-tocopherol primarily among current smokers and the combined group of current smokers and those who recently quit smoking (i.e., within the past 10 years), with a significant test for interaction (Table 5). The latter combined subgroup showed a marginally significant dose-risk trend for serum α-tocopherol. Adding current cigar and pipe smokers yielded similar associations in each of these subgroups; for example, the odds ratio for the highest vs. lowest quintile of serum α-tocopherol was 0.41 (95% CI 0.19–0.90, p-trend = 0.02) in the current smoker-recent quitter category. Prostate cancer risk in the current smoker-recent quitter category was also similar when vitamin E supplement users were excluded: OR = 0.33 (95% CI 0.09–1.24) for the highest quintile (>19.9 mg/L) vs. lowest quintile (≤11.4 mg/L); p-trend = 0.02. When smoking strata were further subdivided by disease severity, the inverse association for serum α-tocopherol among current smokers and recent quitters appeared stronger for aggressive prostate cancer (OR for the highest vs. lowest quintile = 0.24, 95% CI 0.05–1.17, p-trend = 0.06) compared with non-aggressive disease (OR = 0.55, 95% CI 0.17–1.79, p-trend = 0.35). Serum α-tocopherol was not associated with prostate cancer among never-smokers (p-trend = 0.49). For γ-tocopherol, the positive risk association appeared strongest among current smokers and recent quitters, although the tests for trends and the interaction test were not significant.

**Table 5:** Association between baseline serum α-tocopherol and γ-tocopherol and risk of prostate cancer, stratified by smoking status, PLCO Study.

	Serum tocopherol quintiles						
	1	2	3	4	5	p-trend	p-interaction^a^
α-Tocopherol, mg/L	≤12.3	>12.3 & ≤15.0	>15.0 & ≤18.7	>18.7 & ≤24.5	>24.5		0.049
Median, mg/L	10.4	13.8	16.7	20.6	30.6		
Current smokers (n = 46/75)^b^	1.00	2.55 (0.56–11.71) c	0.51 (0.11–2.35)	1.65 (0.31–8.78)	0.51 (0.09–2.83)	0.37	
Current smokers and recent quitters(<10 years ago) (n = 102/144)	1.00	0.93 (0.37–2.35)	0.47 (0.19–1.16)	0.55 (0.21–1.41)	0.39 (0.14–1.04)	0.06	
Former smokers(quit ≥10 years ago)(n = 275/352)	1.00	0.80 (0.46–1.40)	0.91 (0.53–1.59)	0.70 (0.40–1.24)	0.77 (0.43–1.39)	0.44	
Never-smokers (n = 244/247)	1.00	0.62 (0.34–1.15)	0.90 (0.47–1.71)	0.97 (0.50–1.90)	1.02 (0.51–2.05)	0.49	
γ-Tocopherol, mg/L	≤1.38	>1.38 & ≤2.49	>2.49 & ≤3.48	>3.48 & ≤4.78	>4.78		0.28
Median, mg/L	0.96	1.94	3	4.05	5.83		
Current smokers (n = 46/75)	1.00	2.33 (0.32–16.76)	3.68 (0.46–29.36)	1.60 (0.20–13.01)	1.73 (0.24–12.66)	0.80	
Current smokers and recentquitters (<10 years ago)(n = 102/144)	1.00	3.31 (1.09–9.99)	4.65 (1.51–14.36)	1.61 (0.48–5.44)	2.95 (0.91–9.56)	0.55	
Former smokers(quit ≥10 years ago) (n = 275/352)	1.00	1.60 (0.95–2.69)	1.41 (0.83–2.40)	1.32 (0.76–2.30)	1.13 (0.63–2.00)	0.92	
Never-smokers (n = 244/247)	1.00	0.90 (0.47–1.69)	1.69 (0.91–3.15)	1.26 (0.67–2.38)	1.29 (0.65–2.59)	0.28	

a Multiplicative interaction tested using the log-likelihood ratio, comparing models with and without an interaction term of tocopherol quintiles crossed with a categorical smoking status variable.

b Numbers are cases/controls.

c Values are odds ratios (95% confidence intervals), based on unconditional logistic regression and adjusted for study center, serum cholesterol, serum β-carotene, age, time since initial screening, and year of blood draw.

Analyses of other selected subgroups relevant to the vitamin E – prostate cancer association showed that the inverse association with serum α-tocopherol was limited to subjects with total vitamin E intake above the median (OR for highest vs. lowest quintile = 0.36, 95% CI 0.20–0.64, p-trend = 0.01) compared with vitamin E intake below the median (OR = 1.63, 95% CI 0.78–3.39, p-trend = 0.42; p-interaction = 0.03). Risk was also significantly lower for men with high serum α-tocopherol in subgroups defined by older age or lower BMI (data not shown), and significantly higher for men with high γ-tocopherol in subgroups defined by lower serum total cholesterol or shorter follow-up time (data not shown); however, these interactions were not statistically significant. No other subgroups we examined indicated risk interactions for either serum α-tocopherol or γ-tocopherol.

## Discussion

Consistent with some prior studies, we found serum α-tocopherol to be inversely associated with prostate cancer risk. This relationship did not differ materially by disease stage, but appeared restricted to current smokers and recently former smokers (p-interaction = 0.049). By contrast, prostate cancer risk appeared elevated among men in all quintiles of γ-tocopherol above the first. Risk was reduced for men who had both high α-tocopherol and low γ-tocopherol concentrations, but was unrelated to the serum tocopherol molar ratio.

Our findings are supported by other studies where inverse associations between serum α-tocopherol or supplemental vitamin E and prostate cancer were limited to current or recent smokers [6], [8]–[13] or smokers with aggressive disease [5], [7], [26], including a previous analysis of dietary and supplemental vitamin E in PLCO which found lower risk among current and recent smokers for aggressive prostate cancer only [26]. (See Table S1 for a summary of these studies.) By contrast, a protective association for vitamin E supplement use was only evident among never and former smokers in another cohort analysis [27]. Other studies showed no interaction among smoking, serum α-tocopherol or supplemental vitamin E use, and prostate cancer risk [28]–[32], or non-significant inverse or null associations for serum α-tocopherol overall [33]–[36]. Also, several [5], [7], [10], [13], [26], [27], [32], but not all [12], [28], [29], [31], prior studies found stronger inverse relations for advanced disease, with some indicating this only among current smokers or recent quitters [5], [26]. In the present analysis, the protective association for higher α-tocopherol status was slightly stronger for aggressive prostate cancer. While we did observe inverse associations for α-tocopherol in both vitamin E supplement users and non-users, the association was somewhat stronger in the supplement users, suggesting that the higher attained serum α-tocopherol concentrations in the supplement users were related to the findings (median α-tocopherol was 14.8 mg/L for non-users vs. 24.2 mg/L for users). However, although the exclusion of vitamin E supplement users attenuated the risk reduction observed for higher serum α-tocopherol, and vitamin E supplement use was higher across increasing α-tocopherol quintiles, the risk reduction observed in current smokers and recently former smokers persisted even with exclusion of the vitamin E supplement users. This is consistent with other studies where the prevalence of vitamin E supplement use was low and/or the median serum α-tocopherol concentrations were lower than those in PLCO [7]–[10], [13]. For example, in the ATBC and the Physicians’ Health Studies, median α-tocopherol concentrations were, respectively, 11.6 mg/L and 11.1 mg/L, vitamin E supplements were used by 10% and 8% of the men, and ORs were 0.80 (95% CI 0.66–0.96) and 0.51 (95% CI 0.26–0.98, for smokers with aggressive disease) [7], [10]. This indicates that lower prostate cancer risk for higher α-tocopherol concentrations observed among smokers is not limited to vitamin E supplement users.

We found no clear association for serum γ-tocopherol, although prostate cancer risk appeared elevated for men in all quintiles above the first quintile, and adjustment for α-tocopherol had no impact. Circulating γ-tocopherol has been inversely associated with prostate cancer risk in three cohorts [the ATBC Study, the Washington County, MD Study (CLUE), and among smokers for aggressive disease in the Carotene and Retinol Efficacy Trial (CARET)] [9], [13], [31], [33] of eight cohorts in which it was examined [7]–[9], [13], [28], [29], [31], [33], [34]. The median γ-tocopherol concentration in the present investigation (i.e., 3.0 mg/L) is higher than in previous studies (i.e., 1.0–2.9 mg/L), and while the distribution differs greatly (i.e., higher) from that in the ATBC Study [9], it is fairly similar to that in the CLUE and CARET studies [13], [31], [33]. Similar to our current findings, an inverse correlation between the tocopherols was also reported in the National Health and Nutrition Examination Survey (r = −0.37) [37], which contrasts with a positive correlation in the ATBC Study conducted in Finland (r = 0.51) [9]. This difference could be due to the different food sources of tocopherols in Finland and the United States, or greater vegetable oil consumption and α-tocopherol supplement use in the United States [38]. Given the relatively small number of studies that have measured circulating γ-tocopherol, the inverse relationship between serum α-tocopherol and γ-tocopherol, the suppressive effect of vitamin E supplement use (most of which is α-tocopherol) on circulating γ-tocopherol [39], [40], and the identification of both similar and unique biological activities for the two compounds [38], [41], [42], further study of γ-tocopherol is warranted.

The ATBC Study of male Finnish smokers (n = 29,133, 246 prostate cancer cases) was the first controlled trial to report a significant reduction in the incidence and mortality of prostate cancer in response to daily supplementation with 50 mg (50 IU) of α-tocopherol for a median of 6.1 years [1], [43]. Incidence and mortality were reduced 32% and 41%, respectively, with a 40% reduction in incidence of advanced prostate cancer and no reduction for early stage disease [1] (see Table S2 for a review of the trials described here). A subsequent trial in France, SU.VI.MAX (n = 5,141, 103 prostate cancer cases), reported that daily supplementation with 30 mg α-tocopherol for 8 years (along with other antioxidants in the combination supplement) significantly reduced the incidence of prostate cancer among men with normal PSA at baseline (HR = 0.52, 95% CI 0.29–0.92) [44]. Risks did not differ by smoking status, but only 15% of participants were current smokers. Two cardiovascular disease/diabetes trials of daily α-tocopherol supplementation, the Heart Outcomes Prevention Evaluation Trial (400 IU, n = 6,996, 235 prostate cancer cases) and the Heart Protection Study (600 mg in combination with other antioxidants, n = 15,454, 290 prostate cancer cases), showed no effect on prostate cancer incidence [45], [46]. These trials included approximately only 14% and 25% current smokers, respectively, however. Most recently, two trials of healthy men, SELECT (400 IU α-tocopherol daily for a median of 5.5 years, n = 35,533, 2,279 cases) [2], [3] and PHS-II (400 IU alternate days for a median of 7.6 years, n = 14,641, 1,008 cases) [4], also reported no beneficial effect of α-tocopherol supplementation, while additional follow-up of SELECT showed significantly elevated prostate cancer incidence [3]. These two recent trials also included very few current smokers (only 8% and 4% of participants, respectively). In addition to smoking status, another factor potentially related to the inconsistent findings across ATBC, SELECT and PHS-II is the substantially lower vitamin E dose used in ATBC (50 IU/day with beneficial effects), compared with PHS-II (on average 200 IU/day with no effect), and SELECT (400 IU/day with harmful effects). Interestingly, the different dosages resulted in very similar increases in average on-study blood concentrations (from 11.5 to 17.3 mg/L in ATBC versus 12.8 to 18.4 mg/L in SELECT, for example). Another factor that differed among these trials was the baseline eligibility requirement for normal PSA and digital rectal examinations in SELECT, which resulted in few diagnoses of advanced prostate cancer (only approximately 1.1% of all prostate cancers in SELECT) [2], [47]; i.e., precisely the diagnostic category exhibiting lower incidence in the vitamin E arm of the ATBC Study [1]. Although the PHS-II protocol did not require prostate cancer screening at study entry [4], that trial also observed fewer advanced cases than expected, possibly as a result of greater prostate screening consciousness in that population of U.S. physicians [4], [47]. Given that the protective association for vitamin E supplementation or status also appeared stronger for advanced prostate cancer in several observational studies [5], [7], [10], [27], [32], in addition to the ATBC trial [1], which would be consistent with a tumor growth inhibitory effect, the original null results from SELECT and PHS-II may not be surprising. The follow-up findings of significantly greater prostate cancer incidence in the vitamin E groups in SELECT [3] are singular and difficult to explain given substantial previous research.

Cigarette smokers have increased oxidative stress [48], and although circulating tocopherol concentrations tend not to differ between smokers and nonsmokers [48], [49], smokers have increased rates of α-tocopherol disappearance [49]. Therefore, the stronger risk reduction with higher serum α-tocopherol concentrations among smokers is biologically plausible. Although higher vitamin E status could theoretically lower prostate cancer risk in smokers through its chain-breaking antioxidant or anti-inflammatory functions, experimental data indicating tocopherol and tocotrienol inhibition of cell proliferation, cell adhesion, and protein kinase C activity are more consistent with a reduction in tumor progression [14], [15]. For example, prostate cancer cell line growth is inhibited by α-tocopheryl succinate by suppressing androgen receptor expression, prostate-specific antigen, and cell cycle regulatory elements [50], [51]. In a recent analysis of adult men in the National Health and Nutrition Examination Survey III, strong inverse relations between serum α-tocopherol and testosterone, estradiol, and sex hormone binding globulin (SHBG) were observed in cigarette smokers and those with elevated serum cotinine concentrations [52]. These findings corroborated an earlier report from the ATBC Study showing decreased circulating androgens in male smokers supplemented with α-tocopherol [53] and provide a biologically plausible mechanism for the inhibitory influence in the development of prostate cancer. α-Tocopherol supplementation also decreased vascular epithelial growth factor (VEGF) concentrations, which could reduce prostate tumor angiogenesis and growth [54], [55], but this may not be limited to smokers. Cytochrome P450 (CYP) enzymes and other enzymes responsible for metabolism or activation of carcinogens in tobacco [56] are also involved in the metabolism of vitamin E compounds (e.g., CYP3A4 and CYP4F2) [57]–[60]. Induction or competitive antagonism of these enzymes could be consistent with the present findings of a protective association between α-tocopherol and prostate cancer in current smokers, although this may be more relevant for tumor initiation than for growth inhibition.

Properties of γ-tocopherol that differ from α-tocopherol include reduced hepatic secretion into very low density lipoproteins, resulting from the preferential uptake of α-tocopherol by the α-tocopherol transfer protein; selective inhibition of prostaglandin E_2_ synthesis and cyclooxygenase activity; protection against reactive nitrogen species; and inhibition of prostate cancer cells in vitro [18], [38], [41], [42], [61], [62]. How these might account for the suggested positive γ-tocopherol - prostate cancer risk association is unclear and should be examined in further studies. Alternatively, this could simply reflect the suppressive effect of α-tocopherol supplement use on circulating γ-tocopherol [39], [40], as 80% of vitamin E supplement users were in the two lowest quintiles of γ-tocopherol, including 29% that were in both the highest α-tocopherol and the lowest γ-tocopherol quintile. Adjustment for serum α-tocopherol did not alter the risk estimates for γ-tocopherol, however.

The prospective design of our study and the availability of pre-diagnostic blood samples reduced the potential for an effect of prostate cancer on the serum tocopherol measurements (i.e., reverse causality). The availability of data for numerous prostate cancer risk factors facilitated testing and adjustment for potential confounding, and reduced the likelihood of residual confounding. The large number of prostate cancer cases permitted stratification by several factors, notably disease aggressiveness and smoking status, although the number of current smokers was still relatively small. All subjects were selected from the PLCO Trial screening arm (within which biospecimens were collected) and received annual prostate cancer screening under standardized procedures, thereby reducing the likelihood of differential screening practices and diagnostic bias. Fasting status prior to blood collection was not ascertained, and participants were not specifically instructed to fast. Our analyses are based only on baseline tocopherol measures, although in a subset of controls, tocopherol measures one year later were well-correlated with baseline values (r = 0.58 and 0.80 for α- and γ-tocopherol, respectively), and 15-yr repeatability data indicate correlations of 0.46–0.61 for α-tocopherol and 0.48–0.53 for γ-tocopherol [63]. The prevalent use of supplemental vitamin E in this population (31% among controls) and the relatively high serum tocopherol concentrations allowed a robust examination of the hypothesis, although the follow-up time of 8 years was relatively short compared with some other longitudinal studies.

In conclusion, our data indicate a significant inverse association between serum α-tocopherol and prostate cancer risk, observed primarily among smokers. Serum γ-tocopherol appeared to be directly related to risk, but this association was not statistically significant. Our finding of a stronger association among smokers is supported by previous studies, and suggests a biological mechanism for vitamin E that is particularly beneficial among smokers, possibly related to greater tumor growth inhibition in this population. This, along with other factors described above, may partially explain the inconsistent outcomes from clinical trials of vitamin E and prostate cancer, and suggests that further examination of vitamin E and prostate cancer, with careful attention to smoking exposure, disease screening and stage, and mechanisms, is needed.

## Supporting Information

Table S1 **Observational studies of vitamin E and prostate cancer which stratified on smoking status: selected risk estimates.**
(XLS)

Table S2 **Randomized controlled intervention trials which examined vitamin E and prostate cancer.**
(XLS)
